# Modelling vaccination capacity at mass vaccination hubs and general practice clinics: a simulation study

**DOI:** 10.1186/s12913-022-08447-8

**Published:** 2022-08-19

**Authors:** Mark Hanly, Tim Churches, Oisín Fitzgerald, Ian Caterson, Chandini Raina MacIntyre, Louisa Jorm

**Affiliations:** 1 Centre for Big Data Research in Health, UNSW Sydney, Level 2, AGSM (G27), Sydney, NSW 2052 Australia; 2 South Western Sydney Clinical School, Faculty of Medicine & Health, UNSW Sydney, Sydney, Australia; 3 Ingham Institute for Applied Medical Research, Sydney, Australia; 4 Professor Emeritus, SoLES, University of Sydney, Sydney, Australia; 5 The Boden Initiative, Charles Perkin Centre, University of Sydney, Sydney, Australia; 6 Biosecurity Research Program, The Kirby Institute UNSW Sydney, Sydney, Australia

**Keywords:** Stochastic network models, Queues, Health services research, Vaccination, COVID-19

## Abstract

**Background:**

COVID-19 mass vaccination programs place an additional burden on healthcare services. We aim to model the queueing process at vaccination sites to inform service delivery.

**Methods:**

We use stochastic queue network models to simulate queue dynamics in larger mass vaccination hubs and smaller general practice (GP) clinics. We estimate waiting times and daily capacity based on a range of assumptions about appointment schedules, service times and staffing and stress-test these models to assess the impact of increased demand and staff shortages. We also provide an interactive applet, allowing users to explore vaccine administration under their own assumptions.

**Results:**

Based on our assumed service times, the daily throughput for an eight-hour clinic at a mass vaccination hub ranged from 500 doses for a small hub to 1400 doses for a large hub. For GP clinics, the estimated daily throughput ranged from about 100 doses for a small practice to almost 300 doses for a large practice. What-if scenario analysis showed that sites with higher staff numbers were more robust to system pressures and mass vaccination sites were more robust than GP clinics.

**Conclusions:**

With the requirement for ongoing COVID-19 booster shots, mass vaccination is likely to be a continuing feature of healthcare delivery. Different vaccine sites are useful for reaching different populations and maximising coverage. Stochastic queue networks offer a flexible and computationally efficient approach to simulate vaccination queues and estimate waiting times and daily throughput to inform service delivery.

**Supplementary Information:**

The online version contains supplementary material available at 10.1186/s12913-022-08447-8.

## Background

Mass vaccination programs against the SARS-CoV-2 virus are in operation around the world. There is a clear imperative to vaccinate the global population as quickly as possible: high coverage results in less severe illness and fewer hospital admissions resulting from coronavirus disease (COVID-19) [1–3], minimises expensive and disruptive public health interventions [4–10], and limits opportunities for the virus to mutate, potentially resulting in more transmissible or deadly variants [11, 12]. As at February 2022, ten billion COVID-19 vaccinations have been administered globally, with over 20 million additional doses administered daily [13]. While many high-income countries have already achieved high levels of population coverage there is a large global inequity in vaccine distribution, and many highly populated low-income countries remain under-vaccinated [13]. Booster shots are already being rolled out in many countries and the potential requirement to provide annual shots matched to variants of concern is likely to make population-level COVID-19 vaccination programs a fixture of health care delivery for years to come [14–16].

Vaccines are routinely administered at a wide range of locations including hospitals, general practices, and pharmacies. To facilitate rapid rollout of the COVID-19 vaccine, many health departments also established improvised mass vaccination hubs in repurposed venues such as schools, churches, conference centres and sports arenas [17, 18]. The layout and potential capacity of these different venues have implications for how the vaccination process is organised and one of the many logistical challenges of mass vaccination is ensuring adequate staffing and vaccine stock to meet demand and keep overall queueing times to an acceptable level. Getting this balance right is essential to an efficient and successful rollout [19–24]. Too few staff or too few vaccines may result in onerous waiting times or shortages of doses. Having too many doses on site may be impractical for storage reasons, given cold-chain requirements and the necessity to only reconstitute doses that will be used to minimise wastage.

Regardless of where the vaccine is administered, the process involves a sequence of administrative and clinical tasks, that may include temperature checks on arrival, registration or booking confirmation, health assessment, recording consent, vaccine reconstitution and administration of the vaccine. Planning is essential to avoid bottlenecks resulting in long delays and a negative patient experience. In addition, vaccine demand fluctuates over time and it is important to be able to plan for surge capacity.

The application of computational operational research methods to health care delivery and capacity planning has a long history [25–29]. A recent umbrella review of systematic reviews [30] highlights the diverse range of healthcare applications, including obstetrics [31], radiotherapy [32], surgery [33–35], emergency departments [36–38], community health care [39] and distribution of blood products [40]. These methods have also previously been applied to challenges arising in the context of a pandemic. A simulation approach was used to model and optimise nurse allocation in an emergency department in the event of a hypothetical influenza outbreak [41]. Similar methods were used to inform the delivery of a drive-through mass vaccination clinic which successfully vaccinated almost 20,000 residents against the H1N1 virus over 1.5 days in 2009 [42].

Given this long history and array of applications, it is no surprise that the onset of Covid-19 saw the rapid uptake of simulation methods to inform various aspects of the pandemic response. A discrete event simulation was used to model the process of screening and testing for Covid-19 in India using FlexSim Healthcare software [43]. This analysis was used to identify a bottleneck in the testing facilty patient flow, and evaluate potential alternatives through simulation. In Canada, a hybrid discrete-event and agent based model was used to model thoughput at a drive-through mass vaccination clinic [22]. This model estimated total throughput and average processing and waiting times based on a range of assumptions including staff numbers, service times, vehicle occupancy and available drive-through lanes. Implemented using AnyLogic simulation software, the model includes a web-based user interface [44].

In the United Kingdom, an existing open source tool for modelling patient pathways using discrete-event simulation modelling [45, 46] was adapted to model a Covid-19 mass vaccination hub located in a sports stadium [23]. This model informed the organisation of the site by identifying bottlenecks and under-utilisation of resources in the proposed patient flow. The same model was also used to assess the impact of unplanned disruptions at a second vaccination clinic, including delayed arrivals and staff shortages early in the day [24]. The analysis highlighted that early disruptions resulted in significant congestion with undiserable conditions persisting throughout the day, leading the authors to advise fewer daily bookings so that the system could absorb unplanned shocks, or ensuring additional overflow space to safely accommodate patients during congested periods. The underlying simulation model is implemented in R and includes a user interface which can be downloaded and deployed locally [46].

In this study, we aimed to model the vaccination process using stochastic queue network models to help inform public health planning for the delivery of vaccinations. We developed separate models for larger mass vaccination hubs and smaller GP vaccination clinics and show how these models can be used to simulate the queueing process, plan staffing requirements to avoid bottlenecks and estimate daily throughput given constraints on staff capacity. Our analysis adds to the literature in two important ways. First, we incorporate the vaccine preparation process into the queue network, which is important to consider given guidelines to reconstitute vaccines shortly before administration and use within a fixed timeframe. Second, we provide a free and opensource web-based user interface to our simulation models, which allows others to use this modelling tool without needing any specialised software, installation or subscription.

## Methods

### Queueing theory

Queueing is a ubiquitous phenomenon which we encounter daily at shops, airports, train stations and call centres. Queueing theory is a statistical representation of this everyday process. The most basic queue can be characterised by three components: the rate of arrivals into the queue, the service time, and the number of servers [47]. If arrivals are infrequent, service times are fast, and servers are plentiful (e.g. an ATM on a quiet street) then the total waiting time will be short and the average queue length will be low. If arrivals are frequent, the service time is long or the number of servers too few (e.g. at an airport check-in desk on the first day of holidays) then the average queue length will increase as will waiting times.

Queueing theory offers a way to improve the experience for customers and for servers by modelling the queueing process and guiding the balance between these factors. Models of the queueing process represent arrival and service times as stochastic processes. For example, the number of new customers joining a queue in a given period can be modelled as a Poisson process or, alternatively, inter-arrival times can be modelled as an exponential distribution. The aim is to then estimate characteristics of the queue, such as average (median) waiting time and queue lengths given a fixed number of servers, or to estimate the number of servers required to keep average waiting times at a desired level given likely service times and arrivals. Complex queue networks can be formed by joining multiple simpler queues together, either as a tandem network with an ordered series of queues, or a parallel network, with multiple parallel queues [47, 48].

Other features of queue networks include fork/joins and lags. A fork arises when a single queue bisects into two subprocesses; a join arises when two parallel queues merge. Lags are waiting times that don’t involve a server but nonetheless can also be modelled as a stochastic process.

### Modelling the vaccination process as a queue network

In this analysis we represented the vaccination process as a complex queueing network involving tandem queues, fork/joins and lags. We proposed two distinct queue networks—one for mass vaccination hubs and one for GP vaccination clinics—based on real-world examples of how these different delivery modes have delivered COVID-19 vaccines in Australia. For both queue networks, we specified three baseline models based on low, medium, and high staffing availability. We repeatedly simulated data from each model to estimate staff utilisation and service times and, by calibrating the appointment schedule to keep these two metrics within reasonable limits, we estimated baseline daily throughput for each delivery mode. Finally, we performed two what-if scenario analyses to explore how the different queue networks and staffing capabilities responded to two potential system pressures. The first what-if scenario was to incrementally increase the number of appointments, reflecting capability to scale up daily throughput with the same number of staff. The second what-if scenario was to incrementally decrease available staff, reflecting staff shortages due to illness or an increase in competing demands for staff time.

#### Proposed queue networks

Our proposed queue networks for the mass vaccination hub and GP vaccination clinic differ in the layout of stations and how essential tasks are distributed across these stations. Each stage of the process is serviced by one or more servers—staff who undertake the tasks required for that stage. Patients are serviced by the next available server on a first-come-first-served basis, before moving on to the next station in the network.

**Fig. 1:**
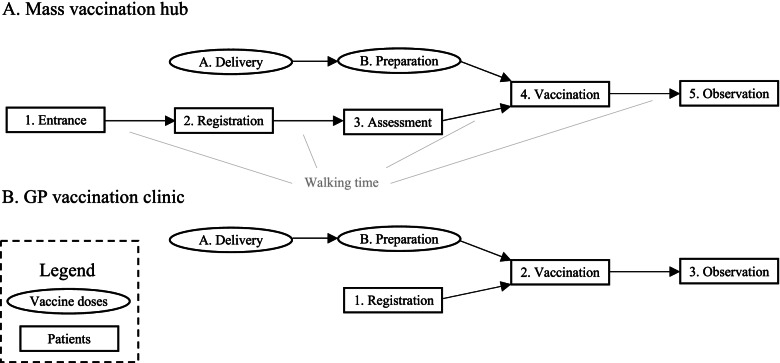
Queue networks for (**A**) mass vaccination hubs and (**B**) GP vaccination clinics

An overview of the two queue networks is presented in Fig. 1 and these are described in more detail below. The main difference is that the GP network—assuming a smaller physical space and fewer staff—distributes the necessary tasks to fewer stations. In this sense, our two proposed layouts are analogous to the ‘Separate’ and ‘Combined’ designs explored by Wood et al. (2021), where the combined design merged the clinical assessment and vaccination stations.

The proposed queue network for a mass vaccination hub was modelled on the Pfizer/BioNTech vaccination hub based at the Royal Prince Alfred (RPA) Hospital in Sydney, Australia. In this queue network, patients traversed five stations: Entrance, Registration, Assessment, Vaccination and Observation. The first four stations require the patient to wait for an available staff member, so these stations are modelled as tandem queueing processes, with new arrivals from the preceding station serviced by the next available staff member on a first-come-first-served basis. The observation station does not require patients to wait for an available staff member but to capture the constraint of observation area capacity this station is modelled as a queueing process where patients are ‘served’ by available seats. Because mass vaccination sites require a large premises, the queue network also incorporated a short walking time between stations. Vaccine doses must be prepared close to the time they are administered, and clearly delays to this process will result in delays at the vaccination stage. To capture this feature of the vaccination process, the queue network includes a parallel queue for vaccine preparation (Fig. 1A) which joins with the patient queue at the vaccination station. The exact steps for the preparation process will vary for the type of vaccine being administered. The assumed activities at each station in the mass vaccination hub queue network are provided in an additional file (Additional file 1 – Appendix A).

The proposed queue network for a local GP vaccination clinic is presented in Fig. 1B. In this queue network, patients traverse three distinct stations: Registration, Vaccination and Observation. To advance through the Registration and Vaccination stations, patients must wait for the next available staff member, so these stations are modelled as queueing processes with patients serviced by the next available staff member on a first-come-first-served basis. As with the mass vaccination model, the observation station is modelled as a queueing process where patients are ‘served’ by available seats and there is a parallel queue specified for vaccine preparation which joins at the vaccination station. Due to the implicit smaller venue size, the time taken to walk between stations at a GP clinic is assumed to be negligible and not included in the model. The distribution of assumed vaccination tasks across the GP queue network is summarised in an additional file (Additional file 1 – Appendix A).

### Parameterising the models

To model queue dynamics based on a given queue networks, three inputs must be provided: (i) simulated services times for each station; (ii) simulated arrival times for each station; and (iii) the number of staff/servers (or open queues) at each station. We defined the service times at each node based on our experience at a mass vaccination hub at the RPA Hospital, Sydney and an exemplar GP clinic. We then calibrated the arrival frequency to ensure baseline models with comparable queue performance for the low, medium and high staffing capacity scenarios using two metrics of queue performance, median processing time and staff utilisation, as described in an additional file (Additional file 1 – Appendix B). The RPA Hospital hub, which had seating for up to 175 patients in the observation area, would constitute a high-capacity mass vaccination hub.

#### Service times

For both the mass vaccination hub and GP vaccination clinic models, the user must specify service time distributions for each station in the queue network. For each patient within the simulation, the time spent at each station is sampled from the corresponding user-specified distribution. In the simulations presented here, the station service times were sampled from exponential distributions with fixed minimum service times and rate parameters as summarised in Table 1. The choice of exponential distributions reflects the assumption that most patients take a relatively short time to process with a minority of patients taking longer.

**Table 1:** Assumed service time distributions for the mass vaccination hub and GP clinic stations

Station	Form	Formula	Percentiles (minutes)				
			5%	25%	50%	75%	95%
**Mass vaccination hub**							
Preparation	exponential	1 + exp.(λ = 3)	1.0	1.1	1.2	1.5	2.0
Entrance	exponential	2 + exp.(λ = 1)	2.0	2.3	2.7	3.2	4.8
Registration	exponential	3 + exp.(λ = 0.7)	3.1	3.4	4.0	5.0	7.3
Assessment	exponential	2 + exp.(λ = 1)	2.1	2.3	2.7	3.4	4.9
Vaccination	exponential	3 + exp.(λ = 1)	3.1	3.3	3.7	4.3	5.8
Observation	normal	norm(μ = 20, 𝜎 = 0.5)	19.8	19.9	20.0	20.1	20.2
Adverse reaction	exponential	20 + exp.(λ = 0.1)	20.4	22.9	26.7	33.0	46.1
**General practice clinic**							
Preparation	exponential	1 + exp.(λ = 3)	1.0	1.1	1.2	1.4	1.9
Registration	exponential	3 + exp.(λ = 1)	3.1	3.3	3.7	4.3	5.9
Vaccination	exponential	5 + exp.(λ = 0.5)	5.1	5.6	6.3	7.7	11.2
Observation	normal	norm(μ = 20, 𝜎 = 0.5)	19.2	19.6	20.0	20.3	20.8
Adverse reaction	exponential	20 + exp.(λ = 0.1)	20.4	22.6	26.6	33.3	48.7

The exception is the observation station, which was modelled as bimodal distribution, assuming normally distributed observation times for the majority of patients who do not experience an adverse reaction and exponentially distributed observation times for a small random subset to reflect a low incidence of adverse reactions. In our simulations the probability of an adverse reaction was set to 2%. The assumed minimum service times and exponential rate parameters for each station are summarised in Table 1, together with the resulting distribution of service times. Our web-based applet allows users to choose from five parametric distributions when specifying service times: exponential, normal, log-normal, gamma and Weibull distributions.

#### Arrival times

Arrivals for both queue networks were generated based on a fixed appointment system for an eight-hour clinic. For mass vaccination hubs we assumed that appointment slots would be given on the hour, every hour. For GP clinics we assumed that appointment slots would be provided in ten-minute intervals. The number of appointments issued per appointment slot for the low, medium and high-capacity scenarios are summarised in Table 2. The number of available appointments was calibrated such that queue performance metrics for our baseline models were within reasonable limits and stable for the three staff capacity scenarios. In particular, the number of appointments was scaled so that the median processing time remained below 60 minutes, and the staff utiliation across all stations remained below 0.8. This calibration process is described in detail in the additional file.

**Table 2:** Assumed arrival frequency for the mass vaccination hubs and GP clinics

Size	Appointment interval (minutes)	Appointments issued per interval
**Mass vaccination hub**		
low	60	60
medium	60	120
high	60	180
**General practice clinic**		
low	10	2
medium	10	4
high	10	6

Stochastic arrival times were generated based on the number of appointments issued per appointment slot with the addition of some random noise, reflecting the assumption that most people arrive somewhat before their allotted time, while a smaller proportion arrive on or after their allotted time. Simulated arrival times also accounted for a small proportion of no-shows, set at 2% for both mass vaccination hubs and GP clinics.

#### Staffing levels

For each of the proposed queue networks we specified models with low, medium and high staffing availability, ranging from 21 to 63 healthcare staff for mass vaccination sites and from 4 to 12 healthcare staff for GP vaccination clinics (Table 3). The distribution of staff across the stations of the queue network was kept stable regardless of the total staffing capacity. For example, for the mass vaccination model there were three staff assigned to the Registration station for every one staff member assigned to the Preparation station, regardless of the assumed size of the hub. This proportionality allows us to compare hubs with different numbers of available staff because the distribution of staff across different stations is consistent.

**Table 3:** Assumed staff numbers by station for low, medium and high staffing availability scenarios

Capacity	Observation area capacity	Staff numbers					
		Preparation	Entrance	Registration	Assessment	Vaccination	Total
**Mass vaccination hub**							
low	25	2	4	6	4	5	21
medium	50	4	8	12	8	10	42
high	75	6	12	18	12	15	63
**General practice clinic**							
low	5	1	NA	1	NA	2	4
medium	10	2	NA	2	NA	4	8
high	15	3	NA	3	NA	6	12

The implied size of the low, medium and high-capacity vaccination hubs and GP clinics is indicated by the corresponding observation area capacity and staff numbers presented above. For example, what we label a low-capacity hub has a total of 21 staff with five vaccinators and 25 seats in the waiting area. A high-capacity hub has 63 staff with fifteen vaccinators and 75 seats in the waiting area. Although the model is agnostic to the physical setting, the former would more closely reflect a local sports hall or community center while the latter would imply a larger venue with more space such as a hospital or stadium.

### Queue performance

We use two metrics to quantify queue performance, processing time and staff utilisation. Processing time, measured here in minutes, is the total time from start to finish of the queue network or equivalently the total time from entrance to exit. Staff utilisation is the average proportion of staff that are serving a patient across the simulation run. An established property of queueing models is that queue performance rapidly degrades as staff utilisation exceeds 80% [49].

### Simulations, software and code

The analysis was performed using R version 4.0.3 and associated packages [50, 51]. Queueing models were simulated using the queuecomputer package, which implements a computationally efficient algorithm that is up to three times faster than tradition discrete event simulation methods [48]. The number of repetitions for each simulated scenario was set to 20, which was adequate to achieve stable estimates of the two queue performance measures. The complete source code to reproduce this analysis is openly available and can be accessed at https://github.com/CBDRH/vaccineQueue.

## Results

**Fig. 2:**
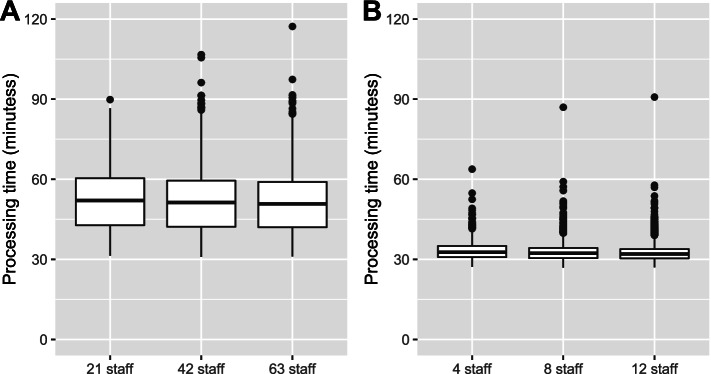
Estimated processing times for (**A**) mass vaccination hubs and (**B**) GP clinics with low, medium and high staffing capacity using the baseline model specifications

For our baseline models, the estimated median processing time at mass vaccination clinics was 52 minutes, and 95% of patients were processed within 67 minutes or less. For GP clinics, the estimated median processing time was 32 minutes, and 95% of patients were processed within 37 minutes or less (Fig. 2). Staff utilisation was kept under 80% for all stations (see Additional file 1 – Appendix B). By design, both measures of queue performance were stable across the low, medium and high staffing capacity for these baseline simulations.

**Fig. 3:**
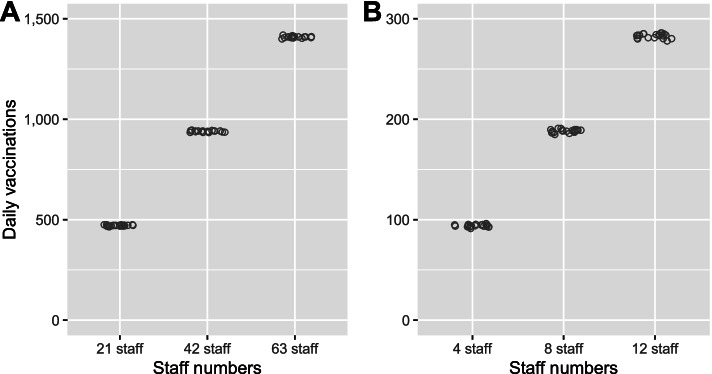
Estimated daily throughput from 20 simulations for (**A**) mass vaccination hubs and (**B**) GP clinics with low, medium and high staffing capacity using the baseline model specifications

The corresponding estimated daily throughput for an eight-hour clinic at a mass vaccination hub ranged from around 500 vaccinations for a low-capacity hub to 1400 vaccinations for a high-capacity hub. For GP clinics, the estimated daily throughput ranged from about 100 vaccinations a day for a low-capacity clinic to almost 300 a day for a high-capacity clinic (Fig. 3). These results show that, while holding queue performance metrics constant, the number of daily vaccinations scales linearly with increasing healthcare staff for both the mass vaccination hubs and GP vaccination clinics.

### What-if scenario analysis

**Fig. 4:**
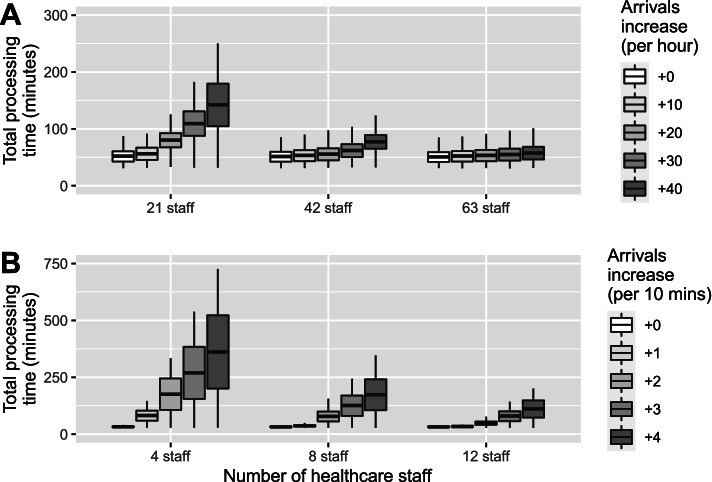
Estimated processing time with increasing arrivals by site size for (**A**) mass vaccination hubs and (**B**) GP vaccination clinics

In this section, we present the results of two what-if scenario analyses. Figure 4 presents the median processing time based on incrementing the arrival frequency from the levels set for the baseline models. The increment step was ten additional arrivals per hour for mass vaccination hubs and one additional arrival every 10 min for GP clinics. In both settings, increasing the number of arrivals resulted in increased processing times. The degree to which sites of different size were able to absorb increased patient numbers varied. For example, at low-capacity mass vaccination hubs (21 staff), an additional 30 arrivals per hour pushed the median processing time to almost 2 h (109 minutes, Fig. 4A), whereas high-capacity hubs (63 staff) were able to absorb this extra patient load while maintaining the median processing time to under an hour (55 minutes, Fig. 4A).

**Fig. 5:**
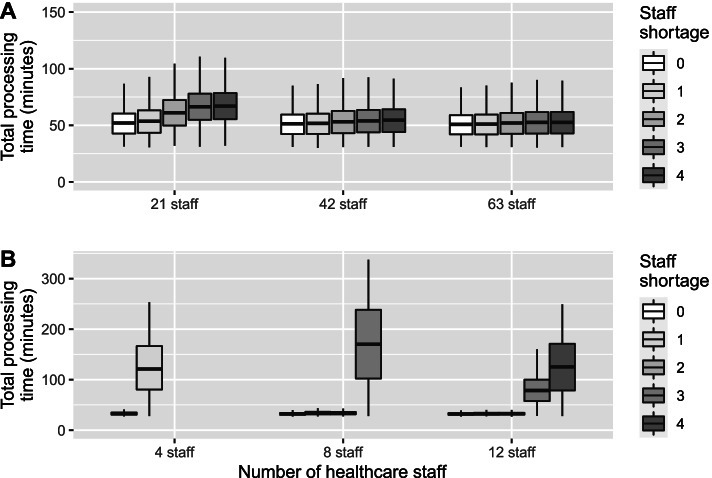
Estimated processing time with decreasing staff numbers by site size for (**A**) mass vaccination hubs and (**B**) GP vaccination clinics

Figure 5 presents the average processing time based on gradually decreasing the available staff for a given model. These results demonstrate that low-capacity vaccination sites with limited staff numbers are quickly affected by staff shortages, whereas large vaccination hubs with more staff can still maintain queue performance with the same number of staff shortages.

### Interactive web-based queue simulation applet

To accompany the analysis presented here, we have developed a free and open access interactive web-based queue simulation applet. This applet provides a graphical user interface to the mass vaccination and GP clinic queueing networks estimated with the R package queuecomputer [48]. On accessing the applet in a web browser, the results from two default models are presented. These models have been parameterised to reflect the medium-capacity baseline model presented here, i.e. the mass vaccination hub with 42 staff members and the GP clinic with eight staff members. The interactive interface allows users to adjust the appointment schedule, the arrival time and service times distributions and the available staff numbers to reflect their own situation or assumptions. Queue performance is summarised in terms of total throughput, processing times and staff utilisation. The applet can be accessed at https://cbdrh.shinyapps.io/queueSim. The underlying source code is openly available on GitHub at https://github.com/CBDRH/vaccineQueueNetworks.

## Discussion

### Summary and discussion of main results

We have used queueing simulation methods to model the vaccination process based on two proposed queue networks emulating delivery at a mass vaccination hub and a GP vaccination clinic. For each setting, we calibrated the number of arrivals that could be vaccinated over an eight-hour period while keeping two queue performance measures—total processing time and staff utilisation—constrained to reasonable levels. Our results provide estimates of potential daily throughput for these distinct vaccine delivery modes across a range of staffing levels. Under our assumed service times, a relatively small GP clinic could perform around 100 vaccinations over an eight-hour clinic, while a relatively large mass vaccination hub could perform around 1400 vaccinations over the same period. Put differently, one large mass vaccination hub can achieve the same throughput as 14 GP small vaccination clinics. GP capacity and feasibility of large-scale hubs may vary by country and geography. In Australia, where there is universal healthcare and thousands of GP clinics, 62% of all vaccine doses have been administered in primary care settings [52].

Our throughput estimates have reasonable face-validity. The mass vaccination hub trialled by New South Wales Health in a 2008 pandemic response field exercise administered 498 vaccines in 5 h using a mass vaccination process delivered through a local school [53]. The Sydney RPA Pfizer hub delivered between 1100 and 1400 daily vaccinations throughout its first month of operation in March 2021.

Our models suggest that daily vaccination capacity scales linearly with staffing capacity while keeping queue performance constant. However, there are several other facets of the vaccine delivery process that are likely to offer economies-of-scale. For example, given a low incidence of adverse events, a high-capacity post-vaccination area observation area could be overseen by a small number of staff members. Economies-of-scale are also likely to apply to vaccine transport, because it may be logistically more efficient and cost-effective to coordinate a single delivery to one centralised hub rather than multiple deliveries to numerous smaller clinics, especially given that the cold-chain must be rigorously maintained at all stages of vaccine transport and handling.

By testing our baseline models using two what-if scenario analyses, we have shown that mass vaccination hubs are better placed to scale up daily throughput with a fixed staff capacity while maintaining acceptable queue performance. We have also shown that mass vaccination hubs are also are more resilient to staff reductions due to absences or some staff being redirected to other urgent duties. Our queue simulation applet provides a simply interface for users to explore the interactions between arrivals, service times and staff capacity. This tool can be used to answer questions such as how many vaccinators would be needed to achieve target daily throughput or how many appointments should be issued given fixed constraints on staff availability and service times.

### Policy implications

Mass vaccination hubs and GP clinics offer distinct advantages as modes of vaccine delivery. As we have shown, mass vaccination hubs are more robust to system pressures like increased arrivals and staff shortages. Smaller GP clinics are more likely to be vulnerable to concomitant, competing workplace demands, which fluctuate during the year and increase notably during the winter months. GP clinics have the advantage of existing infrastructure and existing relationships with patients. GP clinics are also highly flexible and can adapt to local circumstances and specific needs, as seen with carpark drive-through testing sites, which many practices helped set up during the COVID-19 pandemic [54].

The optimal vaccination site may vary for different population segments. Older people or clinically vulnerable patients may benefit from attending their local GP who will be familiar with their medical history. Working adults may benefit from extended hours or more flexible appointment scheduling that could be offered by a mass vaccination hub, as may younger adults—especially among marginalised populations—who are less likely to have a regular GP [55]. It may be easiest to reach university students, and younger children, through vaccination hubs set up in campuses and schools. A combination of larger mass vaccination hubs and smaller GP clinics is likely to achieve mass vaccination faster than either alone.

In practice, the capacity provided by a vaccination site needs to be scaled according to demand, which can vary considerably over time. In NSW, due to an epidemic which started at a time when vaccination rates were low, there was a surge in demand around August 2021, with waning demand as high vaccination rates were achieved. The queue network modelling approach presented here can assist policy makers to plan staffing needs to respond to fluctuating demand.

### Limitations

Our queueing models assume sufficiently available vaccine doses and we have not attempted to model the process of vaccine procurement or the logistics of delivering vaccine doses to the venues where they will be administered. We have proposed two possible queue networks based on our practical experience, but many other configurations are possible. By providing open source code we hope to facilitate others to extend our approach and define their own queue networks. Our analysis does not account for essential staff who are not directly involved in the queueing process but do need to be considered when estimating staffing requirements. For example, the staff needed to oversee the observation station are not included because patients do not have to queue up to be “served” during the post-vaccination observation period. Also not included here are other essential support staff, such as supervisors, cleaners, marshals and caterers. The number and type of support staff required will vary depending on the size and setting of the vaccination hub and must also be considered when planning vaccine distribution. The assumed queue networks rely on subjective assumptions of the distribution of service times at each station. We specified service times distributions that had reasonable face-validity and produced realistic estimates of overall processing times based on our experience, however we did not have resources to formally validate the model results. This could be improved in the future through a time-use survey to empirically measure service times for each station to inform the model inputs as well as total processing time to compare against the model-estimated processing time. Our web-based queueing simulation applet allows queue performance to be explored under different sets of assumptions for service times, appointment schedules and staffing availability.

## Conclusions

Stochastic queue networks can be used to simulate the vaccination process and inform vaccine rollout by exploring the interactions between arrival frequency, service times and staff numbers on queue performance. Different modes of vaccine distribution have different benefits and challenges. Mass vaccination hubs offer a higher daily throughput and are more resilient to increased arrivals and decreased staff availability, however they require larger premises and higher staffing numbers. GP clinics can perform vaccinations at a similar rate per staff member compared to mass vaccination hubs, however it may be difficult to sustain a high throughput given existing workloads. A diverse profile of vaccination sites, drawing on the benefits of both distribution modes, may help to optimise COVID-19 mass vaccination and booster delivery.

## Supplementary Information

**Additional file 1.**

## Data Availability

The datasets generated and analysed during the current study are available in the CBDRH/vaccineQueue GitHub repository at https://github.com/CBDRH/vaccineQueue.

## Abbreviations

COVID-19: Coronavirus disease 2019
GP: General Practice
NSW: New South Wales
RPA: Royal Prince Alfred
