# Supplementary material for: Modelling vaccination capacity at mass vaccination hubs and general practice clinics: a simulation study
Source: BMC Health Serv Res. 2022 Aug 19;22:1059. doi: 10.1186/s12913-022-08447-8 (PMC9388987; doi:10.1186/s12913-022-08447-8)
Supplement: Supplementary file 1 — Additional file 1. [file 12913_2022_8447_MOESM1_ESM.pdf]

Modelling vaccination capacity at mass vaccination hubs and general practice clinics: A simulation study.

Online supplementary material

APPENDIX A. OVERVIEW OF ASSUMED TASKS AT EACH STATION OF THE PROPOSED MASS VACCINATION AND GENERAL PRACTICE QUEUE NETWORKS.

Assumed tasks for the mass vaccination queue network

1. Entrance: Patients arrive at the premises and queue to get a temperature check and check-in to the venue by scanning a QR code with their phone. Hand sanitiser and masks are available. This station is overseen by one or more health professional staff but could also be supplemented with administrative staff to help marshal patients to the next station.
2. Registration: Having passed through the entrance station, patients join the queue for registration. The registration desks are staffed by one or more personnel. As part of the registration process, patients have their current appointment confirmed and can also book their second vaccination, if necessary. They are provided with pre-vaccination information to read while they wait for the next station.
3. Assessment: Once registered, patients join the queue for assessment. The purpose of assessment is to make sure that the patient is clinically suitable to receive the vaccine. During this stage the patient's consent is also recorded.
4. Vaccination: Having been given final clearance to receive the vaccine at the assessment station, patients join the queue for vaccination. Once a vaccinator becomes available, the patient can take a seat and expose their upper arm. The vaccinator confirms the patient's name and details then administers the vaccine. The vaccinator applies a dressing to the vaccination site, notes the vaccination time on a sticker and applies this to the patient's shoulder or lapel.
5. Observation: Once vaccinated, patients advance to an observation area where they take a seat and wait for the required time to ensure they experience no immediate adverse reaction. A staff member

will advise the patient once their observation time has passed, at which point they can exit the premises.

A. Vaccine delivery: The proposed queue network does not set out to model vaccine delivery to the vaccination site. All our analyses assume that an adequate supply of vaccine doses is available at the premises in the quantities required to service all booked patients.
B. Vaccine preparation: Vaccines are delivered in multi-dose vials containing 5-6 doses (Pfizer) or 8-10 doses (AstraZeneca). The exact preparation steps will differ depending on the vaccine being prepared. Steps incorporated at this station may include logging the vial, visual inspection of the dose, reconstitution (for the Pfizer vaccine), and drawing up the vaccine into syringes.

Assumed tasks for the General Practice queue network

1. Registration: Patients arrive at the premises, and receive a temperature check on entry. They are provided with pre-vaccination information and a check-list of contra-indicated items, either as a paper form or on a hand-held tablet. While seated in a waiting area, they read the provided information and complete the pre-vaccination checklist. Once complete, they return the paper form or tablet to the staff member and wait for the next available vaccinator. This process is assumed to take place in a shared waiting area, which may also be used for the observation step.
2. Vaccination: Once a vaccinator becomes available, the patient advances to the vaccination area, which may be a doctor's office or other suitable partitioned area. The vaccinator reviews the patient's pre-vaccination checklist, probes any items that have been checked and records the patient's consent. The patient exposes their upper arm and the vaccinator administers the vaccination and applies a dressing to the vaccination site. Finally, the vaccinator notes the vaccination time on a sticker and applies this to the patient's shoulder or lapel.
3. Observation: Once vaccinated, patients return to the waiting area where they take a seat and wait for the allotted time to ensure they experience no adverse reaction. The waiting area may be monitored by the same staff member who is managing the registration process.

A. Vaccine delivery: The proposed queue network does not set out to model vaccine delivery to the vaccination site. All our analyses assume that an adequate supply of vaccine doses is available at the premises in the quantities required to service all booked patients.

B. Vaccine preparation: Vaccines are delivered in multi-dose vials containing 5-6 doses (Pfizer) or 8-10 doses (AstraZeneca). The exact preparation steps will differ depending on the vaccine being prepared. Steps incorporated at this station may include logging the vial, visual inspection of the dose, reconstitution (for the Pfizer vaccine), and drawing up the vaccine into syringes.

APPENDIX B. CALIBRATING ARRIVALS TO ACHIEVE REASONABLE SERVICE TIMES AND STAFF UTILISATION

In our main analysis we model the vaccination process using stochastic queue network models based on two network specifications, one for a relatively large mass vaccination hub and one for a relatively small General Practice (GP) clinic. For each setting we ran simulations scenarios based on low, medium, and high staffing capacity. The required inputs for these queue network models are the stochastic arrival times, stochastic processing times at each station and fixed staffing numbers at each station in the network. The distribution of service times at each node were defined based on our experience at a mass vaccination hub at the Royal Prince of Albert (RPA) Hospital, Sydney and an exemplar GP clinic. We calibrated the arrival frequency to ensure baseline models with comparable queue performance for the low, medium and high staffing capacity scenarios using two metrics of queue performance, median processing time and staff utilisation. This supplement describes that calibration process.

The number of available staff (and thus the number of open queues) at each station was fixed to be constant at the levels set out in Table 3 of the main text. Within each scenario, the frequency of arrivals was increased incrementally. For example, for the mass vaccination site with low staffing numbers, the frequency of arrivals was increased from 10 per hour to 110 per hour, in steps of 25. For each of the six resulting models, the median processing time and staff utilisation across 20 simulation runs are presented in Figure S1 and S2 respectively.

Figure S1 presents the median processing time as the arrival frequency increases for mass vaccination hubs and general practice clinics with low, medium and high staffing capacity. When arrivals are set to their lowest value, all processing times are within between 30 and 60 minutes, indicated by the vertical grey shaded area. Initially, the incremental increases to the average arrival rate have a negligible impact on the overall median processing time. However, once a certain threshold is reached, the median processing time quickly escalates with each incremental step in the number of arrivals per appointment window. For both mass vaccination hubs and GP vaccination clinics, the critical threshold is lower in the low staffing scenario and higher in the high staffing scenario.

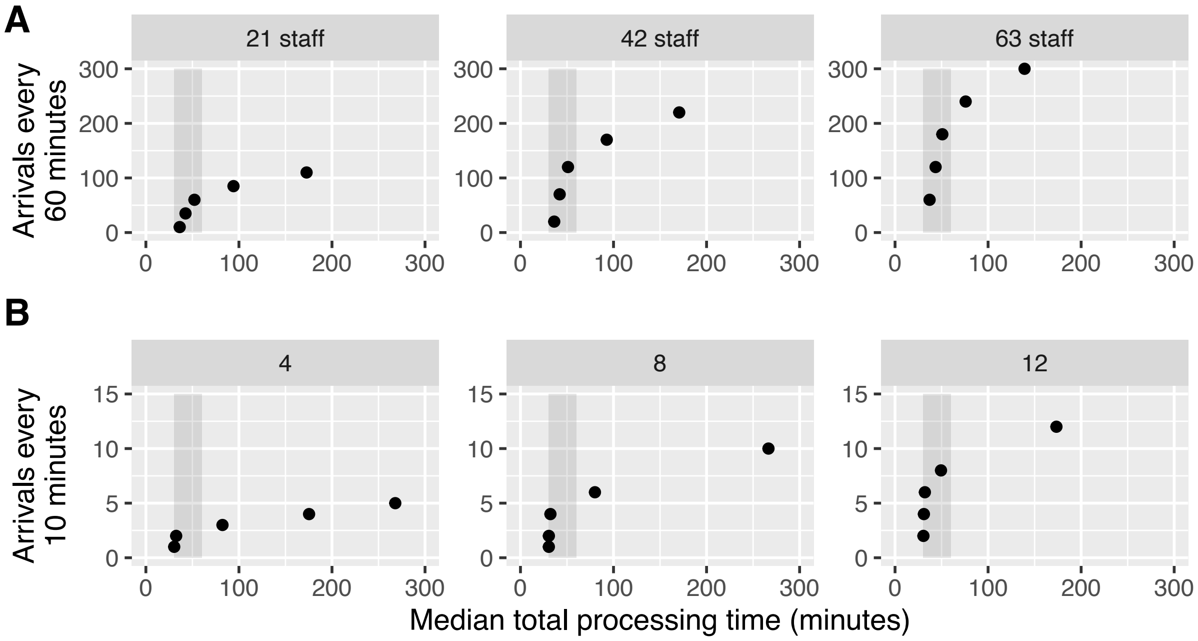

Figure S1. Median processing time as the arrival frequency increases for (A) mass vaccination hubs and (B) general practice clinics with low, medium and high staffing capacity.

Figure S2 presents the corresponding median staff utilisation for the Vaccination station, which was chosen as an example because it is common to both the mass vaccination hub and the GP clinic queue networks. Utilisation for the other stations is not presented but displayed similar patterns. As the arrival frequency increases, mean staff utilisation grows gradually. The vertical grey shaded area indicates a staff utilisation factor between 0.5 and 0.8. Beyond this level, mean utilisation rapidly increases with each incremental step in the number of arrivals per appointment window. These results emphasise the trade-off between arrival frequency, mean staff utilisation and processing times. If arrivals are too low processing times will be at an acceptable level, but the available staff will be under-utilised. As arrivals increase, processing times and staff utilisation increase accordingly. However, if the rate of arrivals grows too high, mean staff utilisation passes a critical threshold and processing times quickly expand beyond reasonable levels. Based on this calibration exercise, we specified the number of arrivals such that the median processing times remained under an hour, and the staff utilisation did not exceed 0.80 for any station.

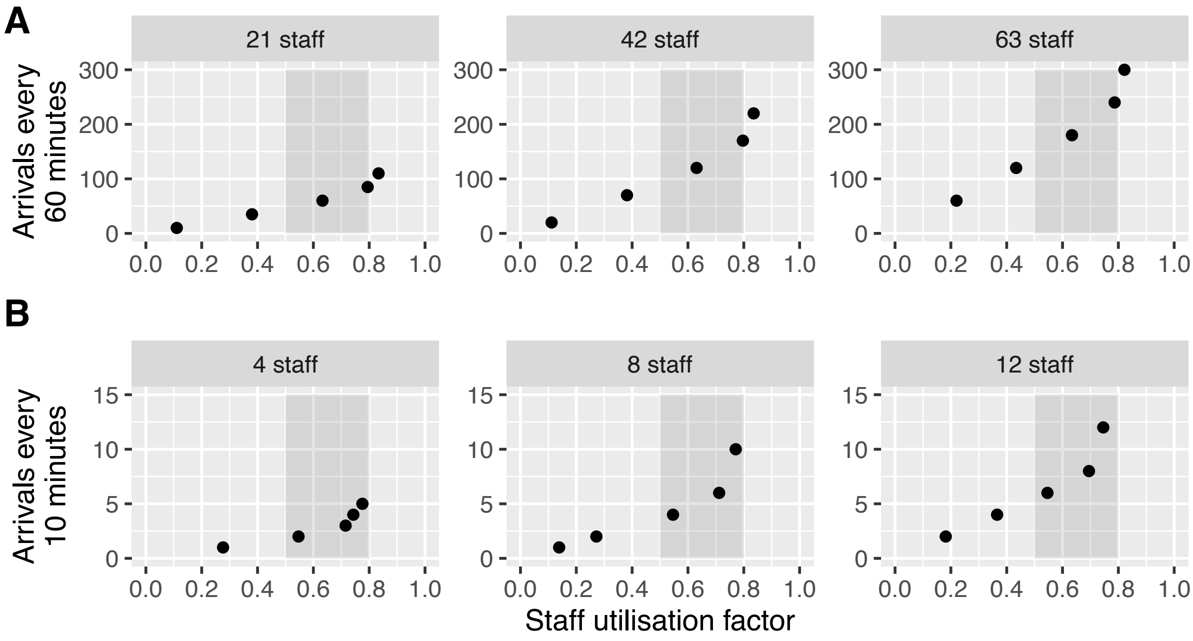

Figure S2. Median staff utilisation as the arrival frequency increases for (A) mass vaccination hubs and (B) general practice clinics with low, medium and high staffing capacity.

The chosen arrival frequencies were selected to increase linearly across the low, medium, and high staffing models: arrivals for the mass vaccination hub were set at 60, 120 and 180 arrivals per hour at relatively low, medium, and high staffed hubs; arrivals for GP clinics were set at 2, 4, and 6 arrivals per 10 minutes (see Figure 3 of the main text). Scaling the arrivals and staffing numbers in this way ensured that the baseline processing times (Figure 2 in main text) and staff utilisation (Figure S3) remained constant across the low, medium and high-capacity simulations within the given queue network. This equivalence facilitates comparisons between hub sizes within the two queue networks. This equivalence is also important for the stress tests reported in the main text because it ensures the different models are starting from the same baseline in terms of queue performance.

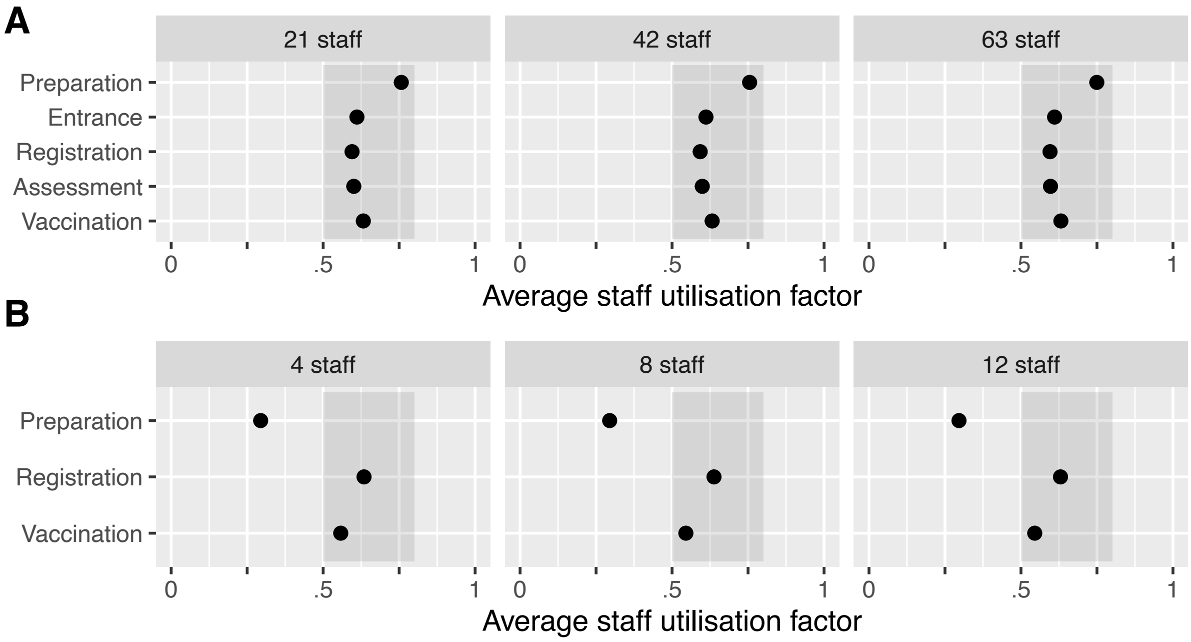

Figure S3. Estimated mean staff utilisation for (A) mass vaccination hubs and (B) general practice clinics with low, medium and high staffing capacity using the baseline model specifications.
